# The Emerging Phenotype of HFpEF in Cardiac Allografts: A Review of Pathophysiology, Diagnosis, and Management

**DOI:** 10.3390/jcm15208130

**Published:** 2026-10-20

**Authors:** Sravya B. Shankara, Sharmini Premananthan, Indranee Rajapreyar, Aaron Sparks

**Affiliations:** 1 Division of Internal Medicine, Tufts Medical Center, Boston, MA 02111, USA; sravya.shankara@tuftsmedicine.org (S.B.S.); sharmini.premananthan@tuftsmedicine.org (S.P.); 2 Division of Cardiology, Tufts Medical Center, Boston, MA 02111, USA; 3 Division of Cardiac Surgery, Tufts Medical Center, Boston, MA 02111, USA; aaron.sparks@tuftsmedicine.org

**Keywords:** cardiac allograft, heart failure with preserved ejection fraction (HFpEF), diastolic dysfunction

## Abstract

Advances in donor selection, organ preservation technologies, donation after circulatory death, and broader geographic sharing have expanded access to heart transplantation but have also introduced new challenges that affect allograft function. The transplanted heart is exposed to multiple insults, including donor brain death, ischemia–reperfusion injury, acute and chronic rejection, and cardiac allograft vasculopathy. Increasingly, heart failure with preserved ejection fraction (HFpEF) is being recognized as a distinct manifestation of cardiac allograft dysfunction that may contribute to late graft failure. Diastolic dysfunction occurs frequently after heart transplantation and may develop early as a consequence of ischemia–reperfusion injury, primary graft dysfunction, and/or rejection. Cardiac allograft vasculopathy, chronic immune-mediated injury, metabolic comorbidities, and aging of the donor heart further contribute to the HFpEF phenotype. Although the process is multifactorial and can be variable, progressive abnormalities in myocardial relaxation, compliance, microvascular function, and extracellular matrix remodeling may lead to restrictive physiology and then the clinical syndrome of heart failure with low cardiac output and end-organ dysfunction despite preserved left ventricular ejection fraction. However, the epidemiology, diagnostic criteria, and natural history of HFpEF in transplant recipients remain incompletely characterized. As a result, the goal of this paper is to consolidate the current understanding of the pathophysiology, epidemiology, diagnostic evaluation, and management of HFpEF in cardiac allografts, while identifying current gaps in understanding to guide future research.

## 1. Introduction

Heart transplantation is the definitive therapy for select patients with end-stage restrictive or dilated cardiomyopathy. Despite advances in transplantation, cardiac allografts develop systolic and diastolic dysfunction. Cardiac allograft dysfunction may occur early (<1 year after transplantation) or late (>1 year after transplantation) and reflects distinct but overlapping mechanisms. Heart failure with preserved ejection fraction (HFpEF) is an increasingly recognized phenotype of allograft dysfunction and is associated with substantial morbidity and mortality. Its pathogenesis is multifactorial and reflects the cumulative effects of donor and recipient characteristics, perioperative injury, denervation, immune-mediated damage, and recipient comorbidities. Allograft injury begins with donor brain or circulatory death, continues through cold and warm ischemia and reperfusion, and may subsequently be compounded by acute or chronic rejection.

In the early postoperative period, ischemia–reperfusion injury and cardiac allograft rejection are principal causes of diastolic dysfunction, manifesting as impaired relaxation and increased chamber stiffness. When these abnormalities persist, they may progress to restrictive allograft physiology and ultimately graft failure. In the late posttransplant period, cardiac allograft vasculopathy, the most common cause of restrictive allograft physiology and late allograft dysfunction, is associated with increased mortality (definitions as per Table 1). Outcomes among transplant recipients who develop HFpEF are worse than those of the overall transplant population. Although HFpEF is emerging as an important phenotype of late graft failure, its natural history, progression, and diagnostic criteria in heart transplant recipients remain incompletely defined. This review summarizes the epidemiology, pathophysiology, diagnostic approach, and management of HFpEF in cardiac allografts.

## 2. Methods

This article is a narrative review based on a structured search of the medical literature to identify evidence on the epidemiology, pathophysiology, diagnosis, and treatment of heart failure with preserved ejection fraction (HFpEF) in cardiac allograft recipients. Databases of peer-reviewed clinical trials, cohort studies, systematic reviews, and professional society guidelines, searching from MEDLINE (via PubMed/Ovid), Embase, and the Cochrane Central Register of Controlled Trials (CENTRAL), were queried from 1980–2026 using combinations of terms including “heart transplantation,” “diastolic dysfunction,” “heart failure with preserved ejection fraction,” “restrictive filling,” “late graft failure,” “left ventricular filling pressures,” and “cardiac allograft vasculopathy,” with attention to incidence, prevalence, risk factors, pathophysiology, and prognostic outcomes. Only original articles in English were included. Retrieved records were screened for relevance to the transplant population, prioritizing studies that evaluated this population, longitudinal cohort studies, randomized controlled trials, and consensus statements/guidelines. If no studies were found including transplant patients, the general guidelines and established randomized controlled trials for the general HFpEF population were analyzed. Because study selection was not performed in duplicate and the search was not exhaustive, this approach is subject to selection bias, and relevant studies may have been missed.

## 3. Epidemiology

Diastolic dysfunction can develop at any time following heart transplantation, with the highest incidence observed during the early posttransplant period. In a single-center study, the incidence of diastolic dysfunction after heart transplantation was 22% at 6 weeks and decreased to 12% at 1 year [8]. Left ventricular diastolic dysfunction (LV DD) was more common than right ventricular diastolic dysfunction (RV DD), but RV DD at 1 year was a strong independent risk factor for cardiac mortality [9]. While the rate of early diastolic dysfunction decreases during the first year posttransplant, HFpEF is a predominant manifestation of late graft failure in this population. Among patients presenting with the clinical presentation of heart failure ≥1 year after heart transplantation (specific diagnostic criteria as noted in Table 2), mortality was 75% over long-term follow-up, 50% had preserved EF on their final echocardiogram, and survival did not differ significantly between those with impaired versus preserved LV systolic function [9].

In a serial invasive hemodynamic study of 17 heart transplant recipients, right atrial and left ventricular filling pressures were elevated during the immediate posttransplant period, despite preserved cardiac output, but normalized by 3 months [10]. Among recipients with normalized resting hemodynamics, volume challenge unmasked occult restrictive right ventricular physiology in approximately half, suggesting persistent subclinical diastolic dysfunction [10]. In another long-term hemodynamic study with more than 5 years of follow-up, left ventricular mass increased throughout the study period and left ventricular end-diastolic pressure remained elevated. In a small cohort of 25 heart transplant recipients evaluated at a median of 8 years after transplantation, 28% met HFpEF criteria at rest, whereas 68% met HFpEF criteria during exercise, independent of donor age and prior rejection episodes. Invasive exercise hemodynamic assessment demonstrated increased left ventricular chamber stiffness, absent contractile reserve, and a rise in left ventricular end-diastolic pressure during exercise [1]. Collectively, these longitudinal structural and hemodynamic changes suggest ongoing allograft remodeling despite an initial improvement in filling pressures. Among a cohort of 547 heart transplant recipients, 32.5% were hospitalized for late graft failure (incidence rate, 3.6 per 100 patient-years), and 60.1% of these patients presented with LVEF ≥ 50% [11]. The most common etiologies underlying late graft failure in this cohort included cardiac allograft vasculopathy (CAV) (50.6%), cellular rejection (44.9%), and antibody-mediated rejection [11]. Furthermore, outcomes in transplant recipients who develop late graft failure with preserved EF are worse compared to those in the general transplant population, with a 5-year retransplant-free survival of 38.4% [11].

Although short- and intermediate-term survival after donation after circulatory death (DCD) heart transplantation is well characterized and appears comparable to that after donation-after-brain-death (DBD) transplantation, longer-term data on CAV and the extent and clinical significance of myocardial fibrosis remain limited. An analysis of the Organ Procurement and Transplantation Network found that the 1-year incidence of CAV was similar after DBD transplantation and DCD transplantation using direct procurement with normothermic machine perfusion; however, CAV occurred more frequently after DCD procurement using thoracoabdominal normothermic regional perfusion [12].

## 4. Pathophysiology

### 4.1. Early Allograft Dysfunction

In this review, HFpEF in cardiac allograft recipients refers to symptoms of heart failure, preserved left ventricular ejection fraction, and objective evidence of elevated filling pressures, in whom rejection and CAV are either absent or are not the main cause of the presentation. Rejection and CAV may either phenocopy or pathogenically contribute to a preserved-EF phenotype and are therefore discussed both as phenocopies, where they account for the presentation, and as overlapping mechanisms, where they act in concert with other processes.

Allograft dysfunction can be classified into early graft dysfunction (<24 h) or subsequent early graft dysfunction (24–72 h) and may manifest as isolated RV, LV, or biventricular dysfunction. Early or subsequent early allograft dysfunction is primarily systolic dysfunction requiring temporary mechanical support or inotropes based on severity [14]. Although consensus definitions focus on systolic dysfunction and do not include diastolic dysfunction as a diagnostic criterion, both systolic and diastolic abnormalities may be present clinically.

The development of the HFpEF syndrome in the early post-heart transplant period is multifactorial and may be influenced by ischemic times, ischemia–reperfusion injury, and the preservation strategy. Both DCD and DBD may expose the donor myocardium to early hypoxic insults that have been proposed to initiate inflammatory signaling and promote subsequent fibrotic remodeling. Donor brain death triggers a catecholamine surge that can cause myocardial necrosis and endothelial dysfunction, with the latter promoting coronary vasospasm and further exacerbating ischemic injury [15]. A prolonged interval between brain death and organ procurement is associated with increased recipient mortality, suggesting that ongoing catecholamine-mediated myocardial injury and endothelial dysfunction in the donor may adversely affect subsequent allograft function and recipient outcomes [16]. The following mechanisms linking organ procurement and presentation to graft injury are biologically plausible but are supported largely by experimental models or by studies of systolic primary graft dysfunction rather than by associations with clinical HFpEF in transplant recipients, for which data remain limited.

During procurement, preservation, and transport, the donor heart is exposed to ischemia, which initiates oxidative stress, cellular injury, inflammation, and potentially residual myocardial fibrosis [17]. Cold ischemic time in DBD and functional warm ischemic time (WIT) in DCD are important determinants of early graft function. In DCD hearts, experimental data suggest that prolonged WIT increases myocardial susceptibility to ferroptosis and pyroptotic cell death [18]. In DBD transplantation, prolonged cold ischemic time, ≥4 h, is associated with increased recipient morbidity and mortality, and this risk is amplified with advanced donor age [19,20]. Prolonged graft ischemic time and advanced donor age are also associated with impaired systolic and diastolic allograft function [21]. Mechanistically, sustained hypoxia disrupts oxidative phosphorylation, shifts cellular metabolism toward anaerobic pathways, and causes cellular edema [17].

Upon reperfusion, the sudden restoration of oxygen can cause transient myocardial stunning [22] and excessive reactive oxygen species (ROS) production, overwhelming endogenous antioxidant defenses [23]. Cellular injury also releases damage-associated molecular patterns (DAMPs), which activate pattern-recognition receptors (PRRs) and initiate inflammatory signaling. The resulting release of proinflammatory and profibrotic cytokines promotes fibroblast activation, myocardial edema, and interstitial fibrosis [24]. Although mild ischemia–reperfusion injury (IRI) may be reversible, severe or prolonged injury can cause irreversible tissue damage, persistent diastolic and, in advanced cases, systolic dysfunction [17]. Serum troponin T measured 3 h after reperfusion correlates with cardiac ischemic time, supporting a direct association between ischemic duration and IRI severity [24]. Residual myocardial edema after moderate IRI may also contribute to increased left ventricular mass and wall thickness during the first 3 months after transplantation [24]. These structural changes may provide a substrate for later diastolic dysfunction, though studies have not established a direct longitudinal link to clinical HFpEF in transplant recipients.

Immune-mediated cellular changes can also occur in the early posttransplant period. Primary graft dysfunction and acute rejection are defined principally by early systolic and hemodynamic compromise rather than by HFpEF physiology; nonetheless, the inflammatory and extracellular-matrix changes associated with these processes may plausibly produce diastolic abnormalities that overlap with or mimic HFpEF. Primary graft dysfunction (PGD) in cardiac allografts is often multifactorial and can result from inflammatory activation after brain death, elevated pulmonary vascular resistance, and IRI [25]. Higher levels of plasma and intra-graft inflammatory markers such as interleukins-6, -8, -10, and -18 have been found in the setting of severe PGD [26]. Acute rejection can be mediated by T-cells or antibodies, due to activation of the recipient immune system against donor antigens in the graft [27,28]. Immune activation can lead to myocardial edema, immune-mediated expansion of the extracellular compartment, and interstitial fibrosis, which may lead to ventricular stiffness, impaired relaxation, and diastolic changes [24]. When immune-mediated injury is the dominant mechanism, the presentation is better classified as rejection-related allograft dysfunction than HFpEF.

### 4.2. Late Allograft Dysfunction

Late allograft dysfunction is multifactorial and reflects the cumulative effects of early graft injury, immune activation, microvascular dysfunction, medication exposure, and recipient comorbidities. These interacting processes promote progressive myocardial remodeling and fibrosis. Among patients undergoing re-transplantation, myocardial fibrosis was significantly more prevalent in those with restrictive physiology than in those without restrictive physiology (94% vs. 15.4%) [29].

Serial endomyocardial biopsies obtained at 6-month intervals for up to 10 years provide insight into the temporal course of this remodeling. Fibrosis was evident at the time of transplantation and progressively increased over 10 years of follow-up [30]. Hypoxic injury at transplantation may activate angiogenic and profibrotic signaling pathways. In turn, fibrosis, endothelial dysfunction, and microvascular rarefaction impair oxygen delivery, which has been hypothesized to establish a self-reinforcing cycle of tissue hypoxia, microcirculatory dysfunction, and adverse remodeling [30,31].

CAV, a major cause of late allograft failure, develops through immune and nonimmune mechanisms. Its pathogenesis involves endothelial injury, vascular inflammation, and fibroproliferation in both the epicardial and microvascular coronary circulation [32]. Recipient T lymphocytes recognize donor human leukocyte antigens, activating cytokine pathways and stimulating the release of growth factors, including platelet-derived growth factor, insulin-like growth factor I, and transforming growth factors α and β. These mediators promote vascular smooth muscle cell migration and proliferation, extracellular matrix deposition, and diffuse concentric intimal hyperplasia [33,34]. Abnormalities in the fibrinolytic pathway and increased expression of endothelial adhesion molecules due to IRI have been implicated in CAV and graft failure. Immune and nonimmune injury also reduce capillary density and increase capillary wall thickness, impairing myocardial perfusion. In severe CAV, the resulting microvascular loss and remodeling appear to contribute directly to diastolic dysfunction and restrictive cardiac allograft physiology, potentially to a greater extent than interstitial fibrosis alone [33,34]. When CAV is the dominant driver of this physiology, the presentation is better classified as CAV-related allograft dysfunction than HFpEF.

Chronic rejection is another important mechanism of immune-mediated myocardial injury and fibroproliferative remodeling that may contribute to a preserved-EF phenotype. Transforming growth factor β (TGF-β) is a central mediator in this process. Its expression is significantly increased in endomyocardial biopsy specimens from cardiac allografts with restrictive filling patterns and is inversely correlated with mitral deceleration time and isovolumetric relaxation time [35]. TGF-β has dual effects in transplantation: although it suppresses alloimmune responses, it also promotes interstitial fibrosis and vascular remodeling during chronic rejection [36]. In antibody-mediated rejection, donor-specific antibodies directed against endothelial human leukocyte antigens activate the vascular endothelium and recruit inflammatory cells, including macrophages, which can amplify TGF-β–dependent profibrotic signaling. Persistent activation of these pathways promotes progressive myocardial fibrosis and adverse remodeling and may overlap mechanistically with the development of cardiac allograft vasculopathy. Furthermore, because these pathways can produce diastolic dysfunction and restrictive filling patterns independent of HFpEF as defined in this review article and require specific therapies, rejection and CAV must be actively evaluated for in every transplant recipient with suspected HFpEF, and their presence should be explicitly documented even when HFpEF physiology coexists.

Calcineurin inhibitors, including tacrolimus and cyclosporine, may further contribute to adverse remodeling through associations with cardiomyocyte hypertrophy and myocardial fibrosis [37]. Hypertension, diabetes mellitus, and chronic kidney disease, common comorbidities for this patient population, further promote allograft dysfunction by inducing endothelial injury, oxidative stress, and chronic inflammation, thereby accelerating microvascular disease and myocardial fibrosis [38].

## 5. Diagnosis

Diagnosing the clinical syndrome of HFpEF in cardiac allografts requires a modified approach from the non-transplant population that integrates clinical findings and resting echocardiography [2,13]. Transplant-specific confounders and competing diagnoses, such as rejection and CAV, which may mimic or contribute to a preserved-EF phenotype, must be actively evaluated and, when they drive the presentation, distinguished from HFpEF. The convergence of the following three elements should be considered when diagnosing HFpEF in transplant patients: (1) symptoms and/or signs of heart failure (exertional dyspnea, fatigue, congestion); (2) a preserved left ventricular ejection fraction (LVEF ≥ 50%), with exclusion of recovered reduced-EF graft dysfunction; and (3) objective evidence of elevated left ventricular filling pressures, demonstrated non-invasively, at rest invasively, or during exercise. An isolated diastolic abnormality or a restrictive pattern, in the absence of all three elements, does not constitute HFpEF. To assess elements 2 and 3, multiple modalities can be used, such as echocardiography and right heart catheterization.

Several conventional echocardiographic indices are less reliable after heart transplantation because donor age, cardiac denervation, and perioperative changes alter diastolic physiology. Resting sinus tachycardia may cause fusion of the mitral E and A waves, whereas perioperative myocardial edema may produce a restrictive-appearing filling pattern with an E/A ratio ≥2 [2]. For heart transplant recipients in sinus rhythm, the 2025 American Society of Echocardiography guidelines recommend a dedicated algorithm based on the average E/e′ ratio, the ratio of mitral E velocity to isovolumic relaxation strain rate (E/SR_IVR_), and peak tricuspid regurgitation (TR) velocity [2]. An average E/e′ <7 suggests normal left ventricular filling pressure, whereas a value >14 supports elevated filling pressure. When the average E/e′ is 7–14, E/SR_IVR_, measured from all three apical views, may help resolve the classification: a value ≤200 cm suggests normal filling pressure, whereas a value >200 cm supports elevated filling pressure. If SR_IVR_ cannot be measured, peak TR velocity may be used as a substitute; a value ≤2.8 m/s favors normal filling pressure, whereas a value >2.8 m/s supports elevated filling pressure. Global longitudinal strain (GLS) may provide additional diagnostic value. In heart transplant recipients, GLS predicted a pulmonary capillary wedge pressure >12 mmHg with an area under the receiver-operating-characteristic curve of 0.932 (*p* < 0.0001) and was more accurate than conventional parameters irrespective of left ventricular ejection fraction or the presence of CAV [13].

Resting hemodynamic and echocardiographic findings may remain normal in cardiac allografts; therefore, exercise testing and/or invasive hemodynamic assessment may be required to unmask clinically significant diastolic dysfunction. As in the nontransplant population, right heart catheterization remains the reference standard for confirming HFpEF. Diagnostic thresholds include a mean pulmonary capillary wedge pressure (PCWP) >15 mmHg at rest or ≥25 mmHg during supine exercise [1,2]. In the broader HFpEF population, cardiopulmonary exercise testing (CPET) combined with exercise echocardiography can help differentiate cardiac, pulmonary, and peripheral contributors to dyspnea based on distinct hemodynamic and metabolic profiles [39]. CPET is especially pertinent after transplantation as it can distinguish central (myocardial diastolic dysfunction) from peripheral contributors to exercise limitation in the denervated graft [40]. CPET may be used as an initial noninvasive screen to determine a cardiac cause of exercise limitation and thereby identify transplant recipients who warrant further study with invasive exercise hemodynamics to confirm elevated filling pressures [41].

While a definitive, transplant-specific algorithm for diagnosing HFpEF has yet to be derived, probabilistic scoring systems for diagnosing HFpEF in non-transplant patients with unexplained dyspnea have been validated and offer a conceptual framework for future approaches to diagnosing post-transplant HFpEF. The H_2_FPEF score is one such system that integrates noninvasive diagnostic criteria (obesity, atrial fibrillation, age >60 years, treatment with ≥2 antihypertensives, E/e′ > 9, and echocardiographic pulmonary artery systolic pressure >35 mmHg) into a weighted score ranging from 0–9 to discriminate HFpEF from noncardiac causes of dyspnea, with the odds of HFpEF doubling for each 1-unit score increase [42]. A related algorithm, HFA-PEFF, employs a stepwise strategy to diagnose HFpEF. After an initial pretest assessment of clinical characteristics (obesity, hypertension, diabetes mellitus, elderly, atrial fibrillation), Step 2 yields a composite echocardiographic and natriuretic-peptide score spanning functional, morphological, and biomarker domains, in which major criteria contribute two points and minor criteria one point, for a maximum of six. A score of ≥5 is considered diagnostic of HFpEF, whereas a score of ≤1 renders the diagnosis unlikely; intermediate scores of 2–4 denote diagnostic uncertainty and warrant further evaluation with diastolic stress echocardiography or invasive exercise hemodynamic testing [43]. It is important to note that neither H_2_FPEF nor HFA-PEFF have been validated in cardiac transplant recipients. Furthermore, both scores depend on echocardiographic parameters that deviate markedly in the denervated, surgically altered allograft, limiting their utility in this setting. A summary of the studies discussed in this review regarding the epidemiology and diagnosis of diastolic dysfunction and HFpEF in cardiac transplant recipients is provided in Table 2.

## 6. Treatment

Treatment of posttransplant HFpEF is derived from the management of HFpEF in native hearts with regard to aggressive risk factor modification, lifestyle management, diuresis, and guideline-directed medical therapy (GDMT). The management paradigm also requires optimizing immunosuppression and managing CAV if present. Medications used to treat HFpEF in transplant patients include sodium–glucose cotransporter 2 inhibitors (SGLT2is), angiotensin receptor blockers/angiotensin-converting enzyme inhibitors, angiotensin receptor-neprilysin inhibitors (ARBs/ACEIs/ARNIs), mineralocorticoid receptor antagonists (MRAs), beta blockers, and diuretics for symptom and volume control. However, evidence for the use of these medications in the cardiac transplant population is derived from general HFpEF trials that have excluded transplant recipients, as well as observational studies of these medications in this population for other indications.

After heart transplantation, SGLT2is may theoretically limit adverse cardiac remodeling through metabolic, renal, vascular, and anti-inflammatory effects. Proposed cardioprotective mechanisms include improved glycemic control, reduced myocardial and renal inflammation, attenuation of sympathetic activity, and correction of anemia through iron mobilization and erythropoietin-mediated erythropoiesis. SGLT2is may also slow the progression of chronic kidney disease related to hypertension, diabetes, and calcineurin inhibitor toxicity, thereby decreasing adverse allograft remodeling. The transplant-specific clinical data are observational and limited to non-HFpEF outcomes. In a retrospective cohort study of heart transplant recipients with diabetes, SGLT2i use was associated with lower risks of hemodialysis, graft rejection or failure, hospitalization, and all-cause mortality [44]. A subsequent meta-analysis of solid organ transplant recipients with diabetes found that SGLT2is had a generally favorable safety profile, with most adverse outcomes, such as urinary tract infections and graft rejection, occurring at rates comparable to or lower than those in the control group [45]. However, volume depletion and genitourinary infection remain concerns with SGLT2 inhibitor use, and individualized monitoring of volume status, renal function, and infectious symptoms is warranted, particularly in patients receiving nephrotoxic immunosuppression. As these findings are derived from observational studies and heterogeneous transplant populations and were obtained in recipients with diabetes for outcomes other than HFpEF, they should not be interpreted as evidence of efficacy for posttransplant HFpEF.

No randomized trials have evaluated mineralocorticoid receptor antagonists (MRAs) specifically for HFpEF after heart transplantation. Current use is therefore extrapolated from the Class IIb recommendation for MRAs in the general HFpEF population, the TOPCAT findings with spironolactone, and the FINEARTS-HF results with finerenone [46,47]. In transplant recipients, particular concerns include hyperkalemia and worsening kidney function, especially when MRAs are combined with calcineurin inhibitors or other immunosuppressive therapies that affect renal function and potassium homeostasis [48]. Although preclinical studies suggested that mineralocorticoid receptor blockade might attenuate calcineurin inhibitor-associated nephrotoxicity, the SPIREN trial in kidney transplant recipients found that spironolactone did not improve kidney function, provide a sustained reduction in proteinuria, or attenuate progression of interstitial fibrosis over 3 years [49].

Angiotensin-converting enzyme inhibitors (ACEis) have been studied in heart transplant recipients primarily for hypertension, renal protection, and prevention of CAV, rather than HFpEF development or outcomes; however, treatment of these comorbidities may nonetheless mitigate adverse allograft remodeling. In an observational cohort, initiation of ACEis early after transplantation was associated with better renal function and lower pulmonary artery systolic pressure at 1 year [50]. In a randomized trial, ramipril did not significantly reduce the long-term composite of death, re-transplantation, hospitalization for rejection or heart failure, or coronary revascularization [51]. However, event rates were numerically lower, and treatment was well tolerated with improved coronary microvascular function [51]. ACE inhibition has also been proposed to reduce CAV and graft rejection by modulating renin–angiotensin–aldosterone system activation and its associated vascular, inflammatory, and profibrotic effects [52]. With ramipril initiated within 8 weeks post-heart transplant, coronary plaque volume was not attenuated at 1 year, but coronary microvascular function improved due to lower microcirculatory resistance and greater coronary flow reserve [53]. Evidence supporting ARNIs, sacubitril/valsartan, after heart transplantation is limited to a single case report in a recipient with reduced left ventricular ejection fraction, in whom treatment was associated with improved LVEF and pulmonary hypertension [54]. Accordingly, its efficacy for HFpEF in cardiac allografts remains unknown. From a transplant-specific safety perspective, RAS blockade carries a risk of hyperkalemia and worsening renal function, which is heightened when combined with calcineurin inhibitors (CNIs) or other immunosuppressants that affect renal function and potassium homeostasis [52].

Beta-blocker use after heart transplantation has historically been controversial because the denervated allograft relies heavily on circulating catecholamines, raising concern that heart-rate reduction could worsen chronotropic incompetence, cardiac output, and exercise capacity [55]. Emerging evidence supports selective use of beta-blockers for established indications, including persistent sinus tachycardia, supraventricular or ventricular tachyarrhythmias, left ventricular systolic dysfunction, and hypertension [55]. Their role in treating HFpEF after heart transplantation, however, has not been adequately studied.

In terms of symptom management, loop diuretics remain the cornerstone of treatment in HFpEF even after heart transplant. However, as with GDMT discussed above, no transplant-specific randomized trials have examined this. One consideration is that, as the cardiac allograft is preload-dependent, it relies on mechanical principles related to the Frank-Starling curve rather than autonomic reflexes [56]. Cardiac denervation also causes a maladaptive fluid response, leading to increased blood volume compared with controls and elevated atrial natriuretic peptide levels [57]. This preload dependence makes the allograft particularly sensitive to overdiuresis, requiring nuanced fluid management with close monitoring and titration to the lowest diuretic doses to maintain euvolemia [58].

As discussed, CAV is a major cause of late allograft failure and may contribute to post-transplant HFpEF. Aspirin and statins are commonly used as part of preventive medical therapy. Because of their antiproliferative effects, mammalian target of rapamycin inhibitors (mTORi), including sirolimus and everolimus, can attenuate CAV progression. Observational data suggest that substituting sirolimus for a calcineurin inhibitor (CNI), particularly within the first 2 years after transplantation, is associated with less plaque progression and may improve survival and freedom from CAV-related cardiac events [59]. Late conversion to mTORi (>2 years) has not been associated with attenuation of plaque progression [32,60]. CNIs may also promote adverse myocardial remodeling through cardiomyocyte hypertrophy, whereas mTOR inhibition has been associated with reduced hypertrophy and improved diastolic function; however, a direct antifibrotic effect in heart transplant recipients remains unproven. Complete CNI withdrawal carries an increased risk of acute cellular rejection, particularly when conversion occurs early after transplantation [32]. Therefore, CNI withdrawal and conversion to mTORi-based therapy should be individualized according to the recipient’s rejection history, renal function, and CAV burden. Lifestyle modification also plays a role in CAV management. Cardiac rehabilitation has been associated with reduced CAV severity, along with hospitalization and death post-transplant, and is therefore recommended for all patients [61].

Chronic kidney disease (CKD) may contribute to adverse cardiac remodeling and is a major complication of long-term calcineurin inhibitor (CNI) exposure. In the SCHEDULE trial, early CNI withdrawal with conversion to an everolimus-based regimen improved measured glomerular filtration rate at 1 year compared with continued cyclosporine therapy, although biopsy-proven acute rejection was more frequent after CNI withdrawal [62]. Belatacept, a selective T-cell co-stimulation blocker, has also been evaluated as a renal-sparing strategy in heart transplant recipients. In a retrospective single-center cohort, belatacept-based therapy was associated with improved renal function without a significant increase in clinically meaningful rejection [63,64]. However, viral infections, including Epstein–Barr virus viremia, remain an important safety concern [63]. These findings are observational and require confirmation in larger prospective studies.

In summary, management of cardiac allograft dysfunction should emphasize early recognition, prevention, and treatment of modifiable comorbidities including hypertension, diabetes mellitus, and chronic kidney disease together with surveillance and treatment of CAV.

## 7. Conclusions

The pathogenesis of post-transplant HFpEF is multifactorial and nuanced owing to the unique physiology of the cardiac allograft. Post-transplant HFpEF remains a clinically important but incompletely defined syndrome, and diagnosing it requires a systematic, stepwise approach that evaluates for allograft rejection and cardiac allograft vasculopathy, which may phenocopy or contribute to the phenotype, and excludes graft systolic dysfunction, pulmonary vascular disease, and noncardiac causes of congestion.

**Figure 1:**
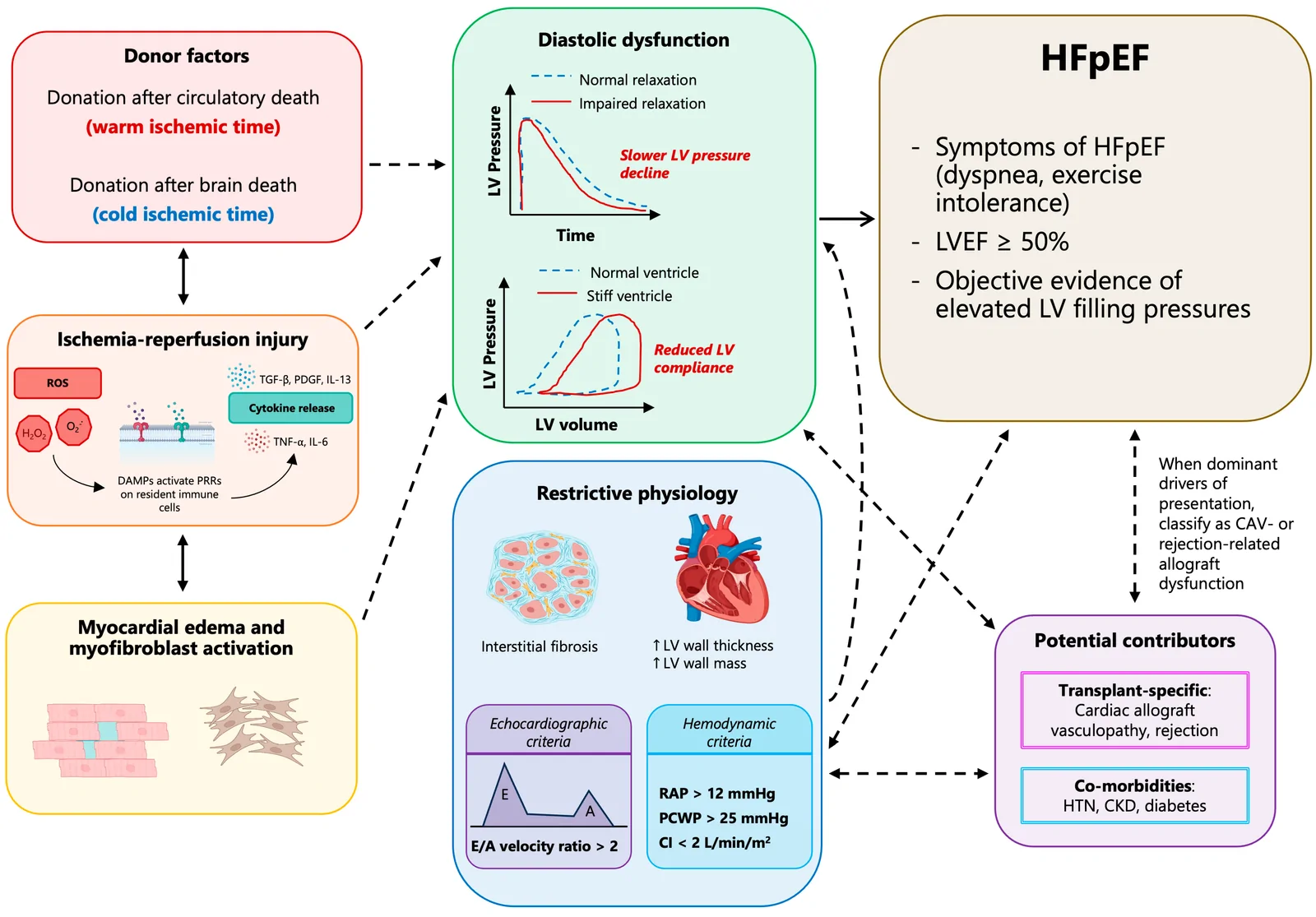
Pathophysiology of posttransplant heart failure with preserved ejection fraction. The development of posttransplant HFpEF reflects a complex interplay of multiple interacting and overlapping pathophysiologic mechanisms spanning cellular injury, subclinical diastolic dysfunction, and the overt clinical syndrome of HFpEF. While restrictive physiology represents the severe end of this spectrum, it is not required for the diagnosis of HFpEF. CI = cardiac index; CKD = chronic kidney disease; DAMPs = damage-associated molecular patterns; HFpEF = heart failure with preserved ejection fraction; HTN = hypertension; IL–6 = interleukin 6; IL–13 = interleukin 13; LV = left ventricle; PCWP = pulmonary capillary wedge pressure; PDGF = platelet-derived growth factor; PRR = pattern recognition receptor; RAP = right atrial pressure; ROS = reactive oxygen species; TGF–β = transforming growth factor-beta; TNF–α = tumor necrosis factor-alpha. Created in part with BioRender.com.

Although early recognition is clinically warranted given the substantial morbidity and mortality associated with this condition, current evidence does not establish that HFpEF-directed treatment improves post-transplant outcomes. Prospective studies of HFpEF in cardiac allografts, including transplant-specific treatment trials, are needed to establish evidence-based management recommendations.(Figure 1)

## Figures and Tables

**Table 1:** Key Terms and the Spectrum of Clinical Pathologies Related to Diastolic Dysfunction, Restrictive Pathologies, and HFpEF. Citations: [1,2,3,4,5,6,7].

Term	Defining Features	Mechanism	Reversibility & Trajectory	Key Distinction
Isolated echocardiographic diastolic abnormality	- Abnormal relaxation indices (septal e′ < 6 cm/s, lateral e′ < 7 cm/s, or average e′ < 6.5 cm/s) ± altered mitral inflow - Without symptoms or elevated filling pressures	- Impaired active relaxation of the LV - Filling pressures are preserved	- Often stable/asymptomatic for years - Does not inevitably progress	- An imaging finding only - Not a diagnosis of HFpEF
Transient early postoperative restrictive filling	- Restrictive-appearing mitral inflow in the early weeks after transplant - EF preserved	- Myocardial edema, ischemia–reperfusion injury, altered loading - Increased chamber stiffness from interstitial/cellular edema	- Reversible - Evolves to a non-restrictive pattern over ~6 weeks as edema and filling pressures resolve	- Time-limited, load- and edema-dependent - Not chronic allograft disease.
Restrictive cardiac allograft physiology	- Heart failure symptoms are common but not required - Echocardiographic and Hemodynamic thresholds used in the ISHLT CAV nomenclature: E/A > 2, IVRT < 60 ms, DT < 150 ms, RAP > 12 mmHg, PCWP > 25 mmHg, CI < 2 L/min/m^2^	- Chronic graft injury: interstitial/replacement fibrosis, persistent immune/inflammatory activation, cardiac allograft vasculopathy - Can occur even without histologic rejection	- Persistent and progressive - Associated with rising PAWP and E/e′ and adverse outcomes - Should prompt evaluation for CAV/rejection	- A chronic allograft-specific phenotype - A severity qualifier within ISHLT CAV classification: ISHLT Grade 3 CAV
Symptomatic HFpEF (with objective elevated filling pressures)	- HF symptoms/signs - LVEF ≥ 50% - Objective evidence of elevated filling pressures (E/e′, LAVI, TR velocity; or invasive PCWP at rest/exercise) ± Elevated natriuretic peptides - Does not require restrictive echocardiographic thresholds or low CI	- Impaired relaxation and increased chamber stiffness raising LV filling pressure - Often with structural remodeling (left atrial enlargement, left ventricular hypertrophy)	- Clinical syndrome - Typically progressive with comorbidity burden	- Requires the clinical syndrome, not just imaging findings - A restrictive Doppler pattern alone is not synonymous with HFpEF
Late cardiac allograft failure with preserved LVEF	- Exertional dyspnea/fatigue years post-transplant with normal resting EF - Marked rise in LV diastolic pressure and PCWP with exercise	- Allograft vasculopathy, microvascular/ endothelial dysfunction, fibrosis, incomplete reinnervation, inflammation	- Late-developing, progressive - Portends graft loss/death	- HFpEF of the allograft

Abbreviations: Ejection Fraction (EF), LV (left ventricle), HFpEF (heart failure with preserved ejection fraction), Cardiac Allograft Vasculopathy (CAV), Isovolumic Relaxation Time (IVRT), Pulmonary Capillary Wedge Pressure (PCWP), Right Atrial Pressure (RAP), Left Atrial Volume Index (LAVI), Tricuspid Regurgitation (TR).

**Table 2:** Summary of Studies Describing Epidemiology and Diagnosis of Diastolic Dysfunction and HFpEF in Cardiac Transplant Recipients.

Study Reference	Design	Sample Size	Transplant Era	Time of Diagnostic Assessment & Follow Up	Definition of Dysfunction/HFpEF	Diagnostic Method	Principal Outcome
Tallaj et al., 2007 [8]	Single-center retrospective study	231 at 6 weeks & 250 at 6 months and 1 year	June 1992–June 2002	- Assessments at 6 weeks, 6 months, and 1 year post-transplant - Follow-up through ≥1 year post-transplant	- LV DD: PCWP ≥ 18 mmHg - RV DD: RAP ≥ 15 mmHg &/or RAP/stroke-volume ratio - Above findings with normal systolic function by echocardiogram and without severe mitral or tricuspid insufficiency	RHC	- Incidence of any DD, LV DD, and RV DD - Cardiac Death
Ambrosi et al., 2016 [9]	Single-center prospective observational cohort study	88 (44 patients who developed heart failure post-transplant and 44 controls)	May 1994–May 2014	- Assessment >1 year post-transplant (at development of heart failure) - Follow-up to 14 years	- Symptoms associated with either LVEF < 50% &/or PCWP > 15 mmHg or mean RAP > 8 mmHg	Rest TTE &/or RHC, respectively	- Death (cardiac, cancer-related, other cause), Heart re-transplantation - Hospitalization for HF - ACS & PCI - Ascites & Dialysis
Young et al., 1987 [10]	Single-center prospective observational cohort study	20 (17 heart transplant recipients & 3 lung)	1980s	- Serial assessment from immediate post-op period onward - Follow-up through >3 months post-op	- Elevated RAP, RVEDP, PCWP - Restrictive RAP patterns & early evidence of tricuspid insufficiency	Serial RHC ± rapid volume infusion & TTE	- Filling pressures on RHC - Restrictive RAP patterns on TTE
Vejpongsa et al., 2022 [1]	Single-center prospective observational cohort study	25	July 2001–January 2015	- Assessment and follow-up immediate post-op till median ~8 years	- LV EDP > 16 mmHg or τ > 48 ms at rest - End-expiratory LVEDP > 23 mmHg - Exercise LVEDP/CO slope > 2 mmHg/L per min - Noninvasive H_2_FPEF score of >2	RHC & TTE	- LV DD at rest and with exercise - Relation between biomarkers and LV DD
López-Sainz et al., 2018 [11]	Single-center prospective observational cohort study	547 recipients	1991–2014	- Assessment from post-discharge onward (at first hospitalization for graft failure) - Mean follow-up of 8.4 ± 6 yr	- First hospitalization for graft failure with LVEF ≥ 50%	Clinical/ hospitalization data, TTE, RHC, biopsy	- Cardiac allograft vasculopathy, cellular rejection grade ≥1R, and antibody-mediated rejection grade ≥1 - Re-transplant-free survival 1, 5, 10 and 15 years after the diagnosis of late graft failure
Bakhtiyar et al., 2026 [12]	Retrospective registry analysis	12,060	December 2019–January 2024	- Assessment and follow-up within the first post-transplant year	CAV incidence over first year (registry-defined)	Organ Procurement and Transplantation Network (OPTN) registry data	- Development of CAV in first posttransplant year
Colak et al., 2020 [13]	Single-center prospective cohort study (with retrospective pre-transplant comparison)	50 OHT recipients (74 paired studies)	Multiple Studies prior to 2020	- Assessment and follow-up variable (at time of endomyocardial biopsy)	- Elevated LV filling pressures: PCWP > 12 mmHg	Speckle-tracking GLS and strain rate on TTE vs simultaneous RHC	- Association of TTE parameters with elevated LVFP
2025 ASE guidelines (Nagueh et al., 2025) [2])	Society guideline/expert-consensus recommendation	Not Applicable	Not Applicable	Not Applicable	- Time constant of LV relaxation (τ) > 48 ms - LV chamber stiffness constant >0.015 - Rest mean PCWP > 15 mmHg - Rest LVEDP ≥ 16 mmHg - Exercise mean PCWP ≥ 25 mmHg - Exercise LVEDP ≥ 23 mmHg -PCWP/CO slope during supine exercise >2 mmHg/L/min	TTE	- Assessing LV diastolic function and diagnosing HFpEF by echocardiography

Abbreviations: Left Ventricular Diastolic Dysfunction (LV DD), Right Ventricular Diastolic Dysfunction (RV DD), PCWP (Pulmonary Capillary Wedge Pressure), Right Atrial Pressure (RAP), Right Heart Catheterization (RHC), Left Ventricular Ejection Fraction (LVEF), Right Ventricular End Diastolic Pressure (RVEDP), Left Ventricular End Diastolic Pressure (LVEDP), Cardiac Output (CO), Transthoracic Echocardiography (TTE), Acute Coronary Syndrome (ACS), Percutaneous Coronary Intervention (PCI), Cardiac Allograft Vasculopathy (CAV), Global Longitudinal Strain (GLS).

## Data Availability

No original data were created or analyzed in this study. Data sharing is not applicable to this article.
